# Enhancing Ion Emission: Insights from Molecular Dynamics and Monte Carlo Simulations

**DOI:** 10.1021/acs.jpclett.4c03640

**Published:** 2025-03-11

**Authors:** Michał Jakub Kański, Soukaina Louerdi, Zbigniew Postawa

**Affiliations:** Smoluchowski Institute of Physics, Faculty of Physics, Astronomy and Applied Computer Science, Jagiellonian University, Łojasiewicza 11, 30-348 Kraków, Poland

## Abstract

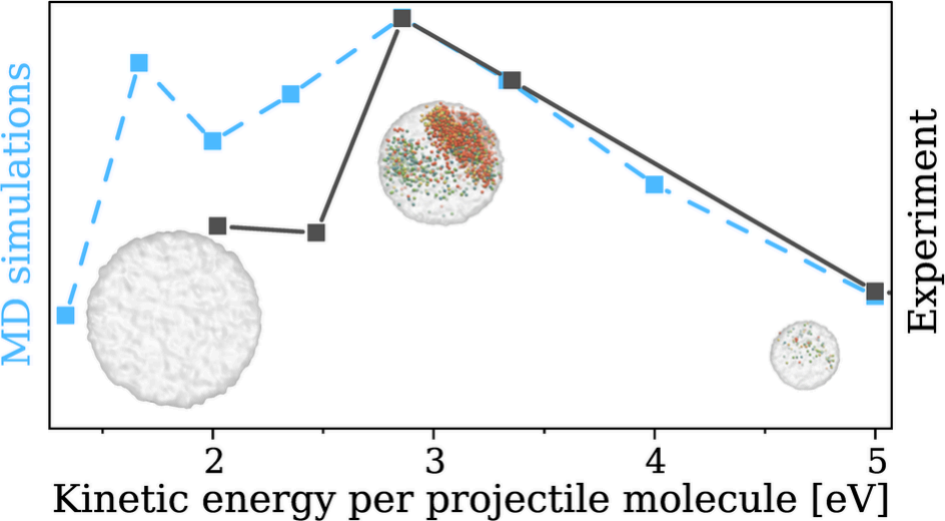

Gas cluster ion beam (GCIB) guns have found several applications in science and industry, such as surface smoothing or depth profiling and surface analysis in conjunction with secondary ion mass spectrometry (SIMS). The former application is severely hindered by the low amount of ejected secondary ions, which can be boosted by more than an order of magnitude by properly selecting the size of cluster projectiles and changing their constituent particles from argon to water. The mechanism of this phenomenon is still unknown. By combining molecular dynamics (MD) and Monte Carlo (MC) simulations with experimental results, we posit that the increase in ion yield can be attributed to proton transfer in long-lived complexes of sample molecules and hydronium (H_3_O^+^) ion from the projectile. The number of molecule–water complexes formed in simulations is directly proportional to the experimental signal intensity, with a small deviation for projectiles containing more than 7000 water molecules.

Surface analysis and depth profiling of organic and, in particular, biological systems require analytical techniques with high spatial resolution and high sensitivity. One of the few methods with such capabilities is secondary ion mass spectrometry (SIMS) equipped with a gas cluster ion beam (GCIB) gun. Such a combination allows for gentle erosion of a sample layer by layer without damaging the underlaying material. Recent breakthroughs in the GCIB design, facilitated by replacing argon atoms with water,^1^ allowed imaging biological structures in much more detail, revealing, for example, additional details in the brain structure.^2^

The enhanced sensitivity comes from the increase in the probability of ionization when massive water clusters (H_2_O)_*n*_ were used. They were shown to increase the ion yield by up to 2 orders of magnitude compared to Ar_*n*_^+^ clusters of a similar size.^3^ Surprisingly, this effect is present only for a very narrow range of kinetic energy per projectile atom, spanning from 2 to 5 eV per water molecule, with a maximum at about 3 eV/mol. Additionally, the enhancement strongly depends upon the type of analyzed solid.^3^ The water clusters appear to be most effective in the analysis of carbohydrates, such as trehalose, while other bio- and organic molecules tend to gain less from changing the projectile from Ar_*n*_ to the (H_2_O)_*n*_ clusters.^3^ The mechanism of the enhancement of the ion yield remains unknown. One proposition states that the increase in ionization probability, especially for molecular ions with an additional attached proton ([M + H]^+^), is a result of an increase in the number of protons and hydroxyl ions created from water molecules broken by impact.^1^ This mechanism is supported by the experiments with the (D_2_O)_*n*_ beam that showed that the additional proton indeed originates mostly from the water cluster projectiles.^3^ However, an enhanced signal is also present for [M – OH]^+^ (molecular ions without a single hydroxyl group) and negative ions, which cannot be explained by this mechanism. Contrary to the above hypothesis, Sheraz et al. speculated that H_2_O molecules will not decompose during an impact because the bond between oxygen and hydrogen is strong (about 5 eV).^1^

In this work, we used reactive molecular dynamics (MD) computer simulations to model trehalose bombarded by (H_2_O)_*n*_ projectiles, where *n* = {25 000, 18 000, 15 000, 12 000, 10 000, 8500, 7000, 6000, 5000, and 4000}, with the same total kinetic energy equal to 20 keV. We show that the experimental signal enhancement is directly proportional to the number of ejected (single trehalose)–water complexes in simulations. This hypothesis is an extension of our previous letter.^4^ Here, we combine MD modeling with Monte Carlo (MC) simulations to provide a plausible explanation for the effect of ion yield enhancement induced by massive water cluster projectiles.

The water cluster projectiles used in this study induce the ejection of material from the sample surface in a very gentle way. Even in the case of (H_2_O)_4000_, only 12 trehalose molecules, on average, decomposed into fragments, usually by the scission of the C–O bond between sugar rings. In the case of (H_2_O)_7000_, only three trehalose molecules undergo chemical reactions. The molecules remain undamaged for impacts of larger clusters. Additionally, not even a single water molecule from the projectile decomposes, even in the case of the (H_2_O)_4000_ cluster. This confirms the proposition of Sheraz et al. stating that the high hydrogen–oxygen bond energy in the water molecule will make its fragmentation improbable.^3^ It is possible, however, that the surface roughness may induce some fragmentation of the projectile molecules.^5^ We plan to conduct a separate study dedicated to this topic in the future. However, the lack of chemical decomposition of both the projectile and the sample suggests that the experimental signal enhancement is not connected to an increased number of free protons created during a projectile impact.

The size of a projectile or equally the kinetic energy per molecule has a tremendous effect on the qualitative aspects of the mass spectrum. The results of our MD simulations of this aspect are shown in Figure 1. It shows how the composition of the sputtered material depends upon the kinetic energy per projectile molecule. The detailed mass spectra can be found in the Supporting Information. The total sputtering yield is linearly decreasing when the size of the projectile increases in the 4000–18 000 range. The smallest cluster projectile induces an ejection of the highest number of trehalose fragments (green stars) as well as single (light blue circles) and clustered multiple (orange triangles) intact trehalose molecules. Almost all of the material is sputtered without adjoining water molecules. Increasing the size of the projectile leads to a shift in the composition of the sputtered material toward the ejection of large clusters of multiple trehalose molecules and water (dark blue diamonds). In comparison to our previous study,^4^ we increased the simulation time for sputtered molecules from 75 to 900 ps. In that time, some (single trehalose)–water complexes (inverted violet triangles in Figure 1) disintegrated into their component molecules without any chemical reactions.

**Figure 1:**
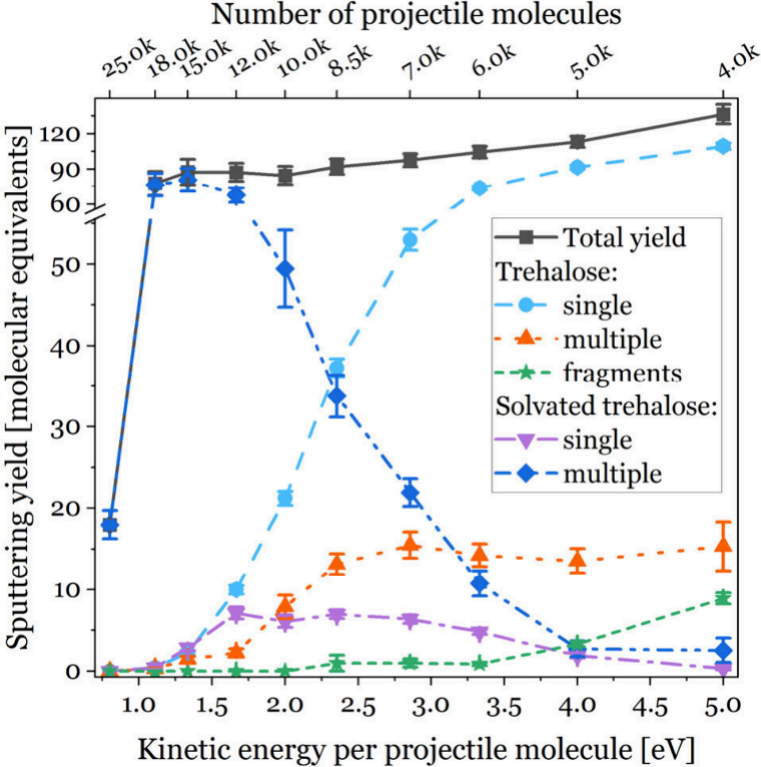
Composition of the total yield (black squares) divided into the following categories: single (light blue circles) and multiple (orange triangles) trehalose molecules ejected without adjoining H_2_O, single (purple inverted triangles) and multiple (dark blue diamonds) solvated trehalose molecules, and molecular fragments (green stars). The lines are used as a guide for the eye. The yield is expressed in multiples of trehalose mass (342.3 amu). The data are the mean of nine separate MD simulations, with the exception of results for projectiles with 5000–8500 water molecules, where 25 simulations were performed.

The decreasing total sputtering yield can be explained in terms of the kinetic energy density transferred to the bombarded system during an impact. The smallest projectile transfers its kinetic energy to a small number of sample molecules. These trehalose molecules acquire a large amount of kinetic energy, which is enough not only for their ejection but also for their fragmentation, as seen in Figure 1. When the size of the projectile increases, its kinetic energy spreads over a larger impact area, decreasing the mean value of the kinetic energy transferred to the trehalose molecules. The fragmentation rate decreases, but a smaller number of molecules gain kinetic energy, which exceeds the cohesive energy of the molecule to the rest of the system. This leads to a decrease in the total sputtering yield.

Increased projectile size activates ejection of “chunks” of the sample consisting of several trehalose molecules. This effect may be explained in the following way. Ejection of *N* molecules as a single chunk requires overcoming the intermolecular forces between the surface of the cluster being ejected and the rest of the system. The energy expended on this process is proportional to the surface area of the cluster. Conversely, the ejection of the same *N* molecules as single entities requires additional energy used for separating molecules within the cluster.

This is what happens for the smaller water cluster projectiles. As the projectile size increases, the amount of kinetic energy transferred to each molecule decreases, as seen in Figure S4 of the Supporting Information. It is not sufficient to eject the molecules as separate objects; therefore, they are ejected as a group, where the adjacent molecules remain bound by the intermolecular forces. A further decrease in the kinetic energy per projectile molecule spreads the kinetic energy across an even larger area and between a higher number of trehalose molecules. During the simulation, the projectile does not decompose into molecules but rebounds mostly intact from the surface, dissolving a few trehalose molecules in the process.

Figure 2 shows the dependence of the experimental [M + H]^+^ trehalose signal^3^ upon the kinetic energy per molecule (black continuous line). There is a strong correlation between the data and a similar dependence upon the amount of (single trehalose)–water complexes ejected throughout the simulations. Only complexes that were stable for at least 320 ps were included. The influence of this parameter can be seen in the Supporting Information. Both graphs are scaled to their respective maxima, and they are remarkably similar, especially in the case of projectiles of up to 7000 water molecules. This result points to the hypothesis that water cluster projectile signal enhancement comes from substrate molecules that were solvated during the impact and then lost their shells of water molecules after being sputtered.

**Figure 2:**
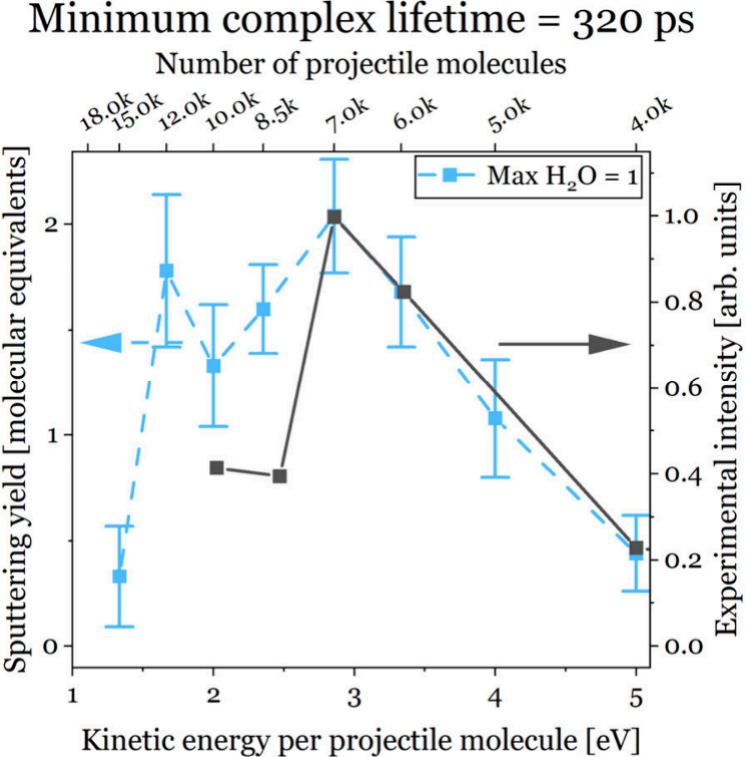
Comparison between the amount of sputtered (single trehalose)–water complexes in MD simulations (blue dashed line) and the experimental signal for trehalose [M + H]^+^ ions (black continuous line) versus the kinetic energy per projectile molecule. Complexes stable for less than 320 ps or containing more than a single water molecule at the end of the simulation are excluded (see the text for an explanation). The lines are added as a guide for the eye.

Why would the presence of such complexes lead to the creation of molecular ions with an additional proton? The kinetic energies involved in the sputtering process are too low to cause substantial ionization through the excitation of a H_2_O molecule. There is, however, a second source of protons; each cluster projectile contains at least one hydronium ion. According to data from the study by Sheraz et al., detecting a single [M + H]^+^ molecule requires about 60/170 impacts in the static/dynamic mode of SIMS, respectively.^3^ Therefore, it is possible that the ions are the result of proton transfer between the initial hydronium ion and one of the sputtered trehalose molecules.

This hypothesis is corroborated by Sheraz et al. experiments with trehalose and dipalmitoylphosphatidylcholine (DPPC) samples irradiated with a (D_2_O)_*n*_ beam. Most of the detected positive DPPC ions and about a half of trehalose cations became deuterated,^3^ pointing to the projectile as the source of the ions’ charge. The remaining positive trehalose ions can be explained by charge transfer between (D_3_O)^+^ and H_2_O in (single trehalose)–water complexes. This H_2_O may originate from the sample preparation, as it was spin-cast from a water solution. Additionally, no deuteration was detected when the surface was bombarded with neutral (D_2_O)_*n*_ clusters and subsequently analyzed with a C_60_ beam. Such a result shows that the cluster projectile needs to be charged and accelerated to achieve signal enhancement.

The chemical structures of massive water cluster projectiles are currently unknown. They are created by supersonic expansion of water vapor into vacuum, which causes aggregation of H_2_O molecules. The clusters are subsequently irradiated by electrons with kinetic energy in the range of 50–150 eV.^1,6,7^ In a simpler case of argon clusters, the impinging electron induces an ejection of the argons’ electron with the lowest ionization energy,^8^ creating a cluster with +1 charge. Conversely, water molecules can decompose as a result of electron loss. Tachikawa and Takada studied the phenomenon of water ionization by *ab initio* molecular dynamics.^9^ They showed that the H_2_O^+^ molecule reacts with a second water molecule to form a H_3_O^+^–OH pair in a matter of several femtoseconds. Furthermore, Li et al. showed that there can be at most only a single H_3_O^+^–OH^–^ pair in neutral liquid water systems smaller than a million molecules.^10^ Combining the information from these studies, we posit that the water cluster projectiles used in SIMS consist of only neutral water molecules, with H_3_O^+^ cations (and accompanying hydroxyl radicals) created by the impinging electron. The actual position of the cation is unknown. The formation of water clusters through the supersonic expansion of gas significantly decreases their temperature to about 215 K.^11^ Such a low internal temperature means that the water cluster projectiles are actually (cryogenic) ice projectiles. In such a cold environment, the proton diffusion is inhibited;^12^ therefore, the hydronium ions should remain close to the ionization point. We studied the ionization probability distribution using Monte Carlo simulations based on the Geant4-DNA framework.^13−17^ Panels c and d of Figure 3 show the results of 1 000 000 electron impacts with a kinetic energy of 100 eV. The probability of ionization quickly decreases with the depth, forming a layer at the surface. The electron impact can lead to heating of the system. On the other hand, hydronium ions are stabilized on the surface;^18^ therefore, random diffusion of the cations should actually lead to their increased concentration on the surface. Additionally, in the case of the (H_2_O)_10 000_ projectile, there is a probability of approximately 30% that the cluster contains at least two hydronium ions (Figure 3e). The occurrence of such clusters increases with their diameter because a longer electron path through the water leads to a higher probability of several ionization events. The excessive charge is balanced by solvated electrons (and the products of their reactions with H_2_O), whose presence can be an explanation for the enhancement of the negative ion signal. The lifetimes of these additional cation–anion pairs are unknown and warrant a dedicated study. However, due to the low temperature of the ice cluster, it should be much longer than in the case of liquid water, studied by Li et al.^10^

**Figure 3:**
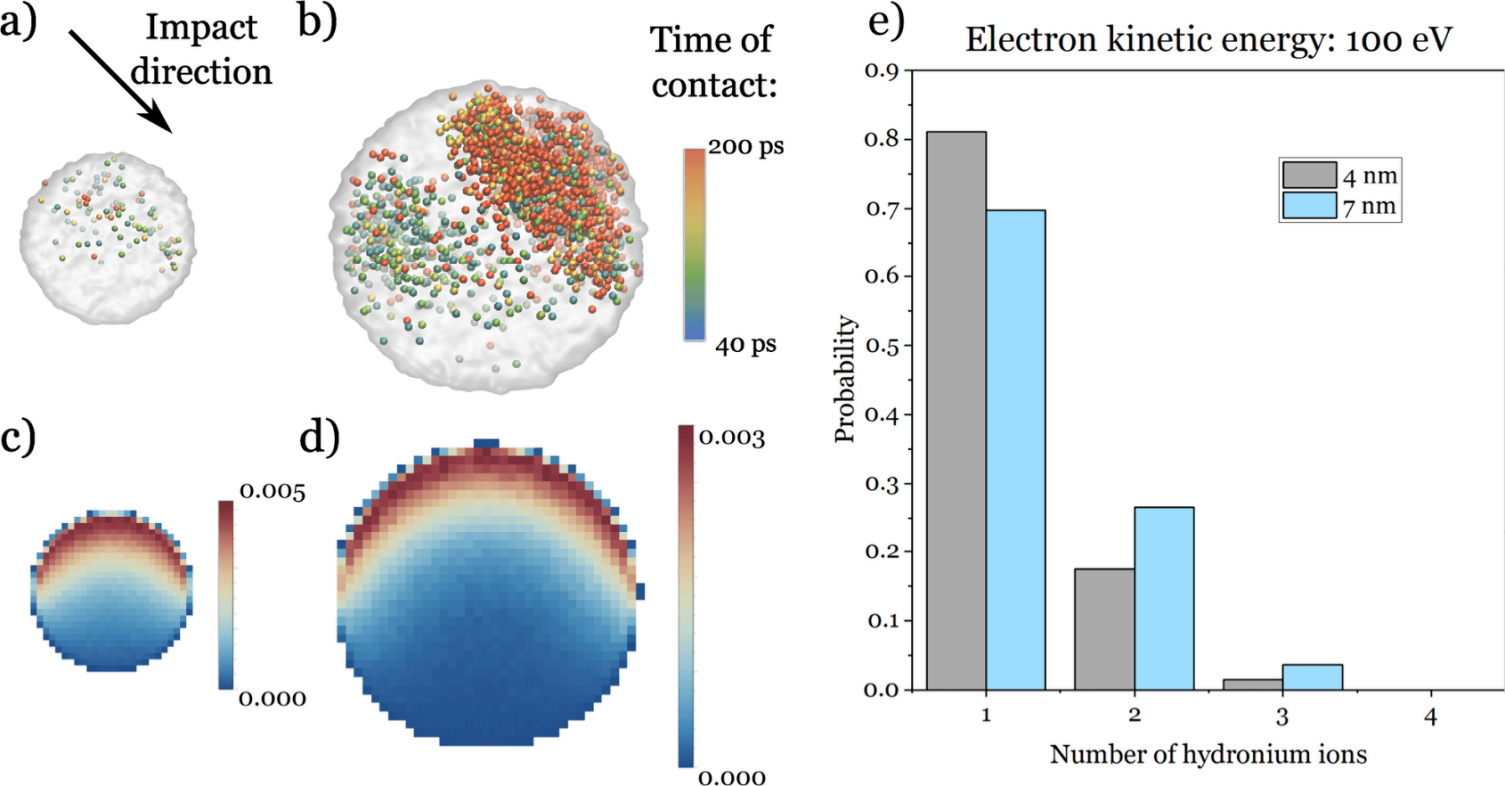
Time of contact between H_2_O projectile molecules and future (single trehalose)–water complexes in the case of (a) (H_2_O)_4000_ and (b) (H_2_O)_10 000_. Only molecules that were in contact for at least 40 ps are shown. Electron ionization probability for singly charged ice clusters with a diameter of (c) 5 and (d) 7 nm, which corresponds to clusters with 4000 and 10 000 H_2_O molecules, respectively. Only 1 nm thick cross sections through the middle of the clusters are shown. Electrons moved downward from the top. (e) Distribution of the number of hydronium ions created by an impact of a single 100 eV electron. The data in the cases of panels a and b and panels c–e are obtained from MD and MC simulations, respectively.

There are three main factors governing the creation of the [M + H]^+^ ions. The most important factor is that the proton needs to be transferred to a close vicinity of a trehalose molecule. Comparing the distribution of hydronium ions to the position of H_2_O molecules that will form (single trehalose)–water complexes (panels a–d of Figure 3) shows that this condition can be met.

Then, a proton transfer reaction must occur, which requires overcoming a reaction barrier. The probability of the reaction is controlled by two major factors: the kinetic energy of the trehalose–hydronium ion system and the time of contact between the molecules. Additionally, Gallo et al. showed that the energy barrier for a proton transfer depends upon the number of water molecules in the vicinity of the molecule to which the proton is transferred.^19^ They calculated that additional water molecules stabilize the proton, which increases the energy barrier from about 0.2 to 1.0 eV when the number of water molecules accompanying the proton increases from 3 to 35. Such a high energy barrier decreases the reaction rate by orders of magnitude. Finally, all neutral water molecules must desorb from trehalose, so that [M + H]^+^ ions can be detected in a mass spectrometer. Because of that, we excluded from Figure 2 (single trehalose)–water complexes where there was more than a single H_2_O molecule at the end of the simulation. Supporting Information contains graphs showing how this cutoff influences the results.

There are several new insights gained from this work. First, because of their low temperature, the water cluster projectiles should be termed (cryogenic) ice cluster projectiles. Our MC simulations show that, during irradiation, hydronium ions are created close to the surface, and their positions coincide with the region of the projectile that forms (single trehalose)–water complexes. Such complexes, after they shed their shell of water molecules, can become [M + H]^+^ ions, as detected in experiments. The remarkable similarity between the experimental data and simulations shown in Figure 2 and the fact that most protons come from the projectile itself^3^ support this hypothesis. More work is needed to understand why the graphs diverge below 3 eV per projectile molecule and why this effect is visible only for some classes of molecules. Calculating proton transfer barriers in molecule–water complexes by *ab initio* methods should provide the necessary information in this regard.

## Computational Methods

A complete description of the MD method can be found elsewhere.^20^ In short, Newton’s equations of motion are numerically integrated using the velocity Verlet algorithm as implemented in the large-scale atomic/molecular massively parallel simulator (LAMMPS).^21^ The time of a single simulation was equal to 75 ps, which was enough to complete most of the sputtering. The trajectories of ejected atoms were further simulated at up to 900 ps. The initial temperature of the sample and impacting projectile was equal to 0 K to speed up the simulations and decrease the number of factors influencing the results. The sample was a hemisphere with a diameter of 60 nm and 134 517 trehalose molecules. Stochastic and rigid zones (with thicknesses of 7.0 and 0.5 nm, respectively) were used to properly contain energy waves originating from the impact.^22^ The density of the trehalose system and water clusters was equal to 1.45 and 1.00 g/cm^3^, respectively. The experimental values are 1.58 g/cm^3^ for trehalose and 0.93 g/cm^3^ for water ice at 77 K.^23^ The modeled and experimental values are within 10%, which is in excellent agreement for reactive force fields. The diameter of the projectiles decreases from about 10 nm for (H_2_O)_25 000_ to 5 nm for (H_2_O)_4000_.

The interactions between atoms were described by the charge-implicit ReaxFF with a tabulated correction, which was created to model high-energy phenomena, such as sputtering.^4^ A variable time step was used during the simulations, which was chosen so that the maximum atom movement during a single simulation step did not exceed 0.005 nm. Some of the simulations were performed using GPU acceleration provided by the KOKKOS package,^24,25^ while others used standard ReaxFF implementation.^26^ The angle of incidence was 45°, a value similar to that used in the experiments. The direction of the projectile was in the *xz* plane, with the *z* dimension denoting the vertical axis. All visualizations were made with Visual Molecular Dynamics (VMD) software.^27^

Molecules were identified as an interconnected network of atoms based on the bond strength (or bond order) calculated in the ReaxFF formalism.^28^ This number varies from 0 to 3 depending upon the distance between the atoms and their chemical environment. For example, the bond order for a double bond is equal to approximately 2. In our analysis, two atoms were considered connected if their bond order exceeded 0.3. Such a value is low enough to take into account bond stretching due to the internal kinetic energy. Clusters are defined as a set of molecules that are in contact. Two molecules were considered in contact if the smallest distance between their atoms was less than 0.3 nm. Clusters that were at least 15 nm above the sample at the end of a simulation and possessed a positive *z* component of center-of-mass velocity were considered as sputtered. The trajectories of such clusters were simulated further, up to 900 ps, to check their stability.

The results of the cluster impact simulation only weakly depend upon the initial coordinates of the projectile.^29^ However, we performed each simulation multiple times with slightly changed initial coordinates of the projectile’s center of mass in order to decrease the statistical uncertainties. The number of simulations performed varied depending upon the projectile size. We performed 25 simulations in the case of 5000–8500 projectiles and 9 simulations for 10 000–18 000 and 4000 projectiles. Only a single impact was modeled for the largest projectile, and the standard deviation was estimated to be equal to 10%.

The ionization probability was calculated by MC simulations with utilization of the Geant4-DNA framework^13−17^ using option 4, which is recommended for modeling the interaction between water and low-kinetic-energy electrons. The simulations take into account processes such as elastic scattering, molecular excitation, and ionization. Electrons with kinetic energies below 11 eV were considered solvated. The sample was a sphere of water (Geant4 material name “G4_WATER”) with a diameter of 5 and 7 nm, representing (H_2_O)_1000_ and (H_2_O)_10 000_ cluster projectiles, respectively. The initial position of an electron was chosen at random. It impacted the sample with a kinetic energy equal to 100 eV. The simulations were run to the point when a million clusters with an overall charge equal to +1 were created.

## Supporting Information Available

Mass spectra, details of calculations of the number of impacts required for detection of a single [M + H]^+^ ion, kinetic energy transfer between the projectile and sample, influence of the minimum lifetime of sputtered (single trehalose)–water complexes, and number of H_2_O in the remaining complexes on the dependence of the number of such complexes upon the kinetic energy per projectile molecule (PDF)
