# Supplementary material for: Enhancing Ion Emission: Insights from Molecular Dynamics and Monte Carlo Simulations
Source: J Phys Chem Lett. 2025 Mar 11;16(11):2875–80. doi: 10.1021/acs.jpclett.4c03640 (PMC11931526; doi:10.1021/acs.jpclett.4c03640)
Supplement: Supplementary file 1 — jz4c03640_si_001.pdf [file jz4c03640_si_001.pdf]

Supporting information

Enhancing Ion Emission:
Insights from Molecular Dynamics and Monte Carlo Simulations

Michał Jakub Kański*, Soukaina Louerdi, Zbigniew Postawa

Jagiellonian University, Faculty of Physics, Astronomy and Applied Computer Science, Smoluchowski Institute of Physics, Łojasiewicza 11, 30-348 Kraków, Poland

* Author to whom correspondence should be addressed

Mass spectra

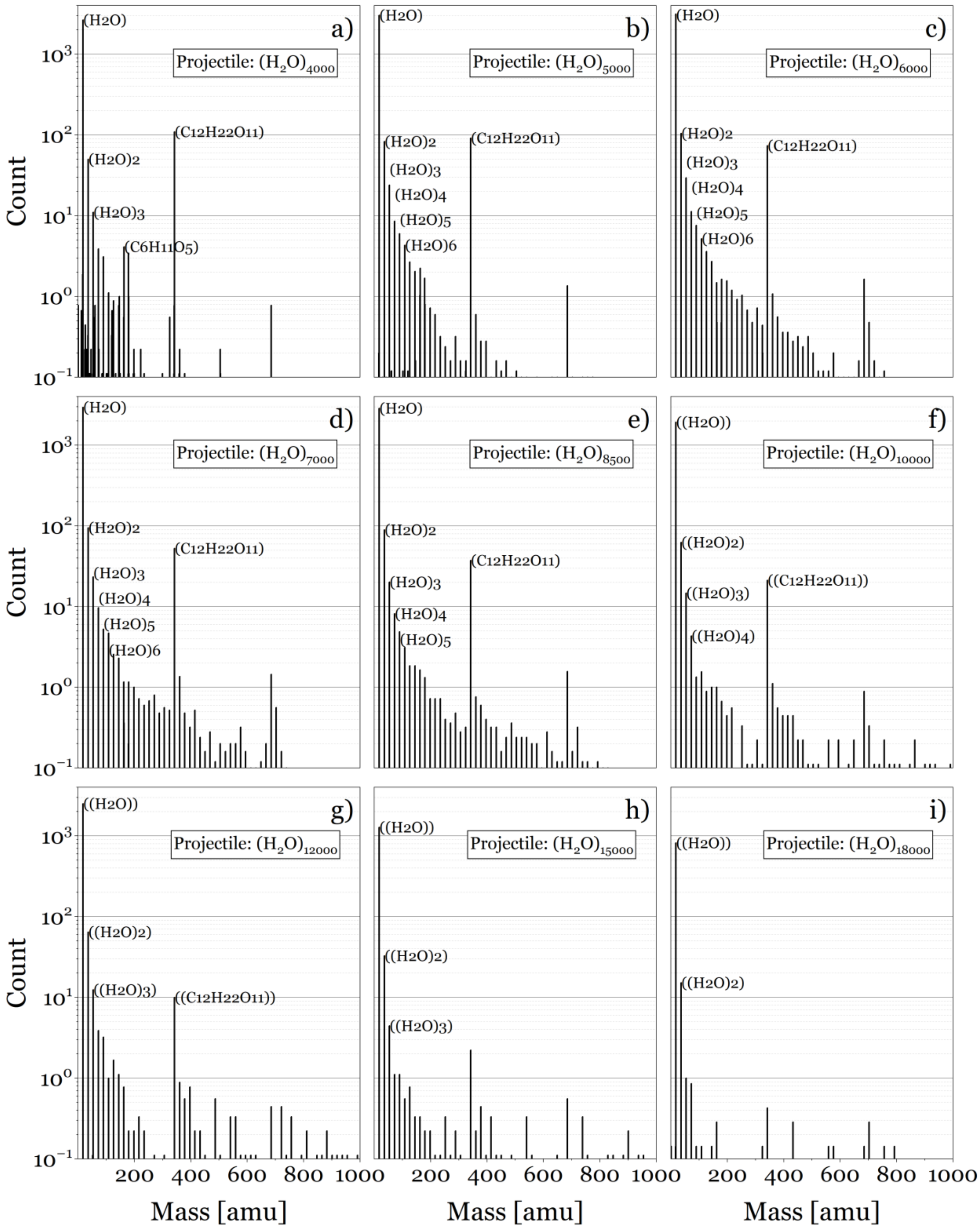

Figure S1 - Mass spectra of trehalose bombarded by $(H_2O)_n$ clusters where n = 4000 (a), 5000 (b), 6000 (c), 7000 (d), 8500 (e), 10000 (f), 12000 (g), 15000 (h), 18000 (i) 900 ps after impact. The data are a mean of 9 (a,g-5) or 25 (b-e) separate simulations. The graphs show the low-mass region (below 1000 amu).

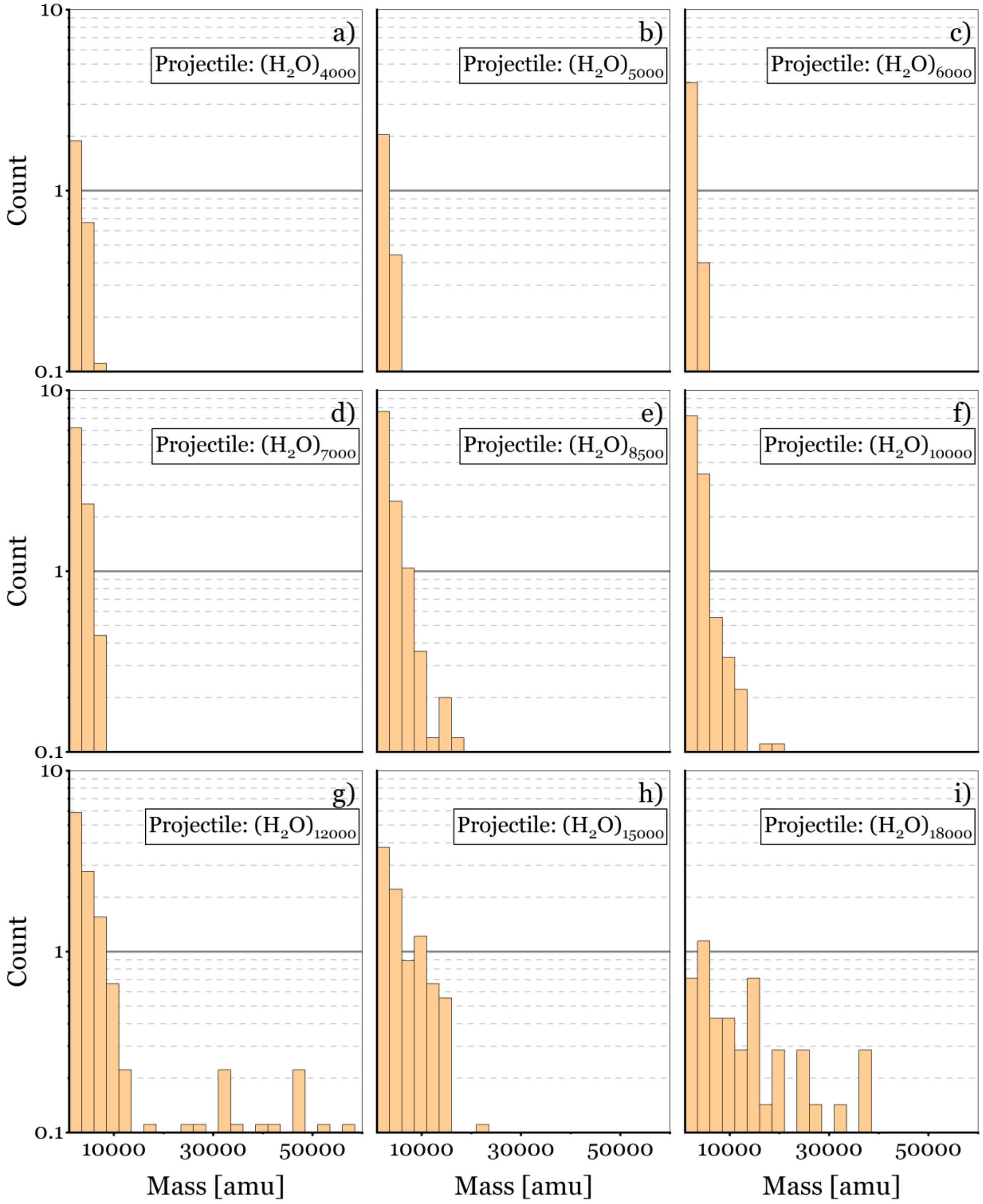

Figure S2 - Mass spectra of trehalose bombarded by $(H_2O)_n$ clusters where n = 4000 (a), 5000 (b), 6000 (c), 7000 (d), 8500 (e), 10000 (f), 12000 (g), 15000 (h), 18000 (i) 900 ps after impact. The data are a mean of 9 (a,g-5) or 25 (b-e) separate simulations. The graphs show the high-mass region (above 1000 amu) with data binned every 2500 amu.

Number of impacts required for detection of a single $[M+H]^+$ ion

Figure S3 shows the mean number of projectiles required to detect a single $[M+H]^+$ trehalose ion in the case of static (ion dose = $5\cdot10^{11}$ ions/cm^2) and dynamic (ion dose = $3\cdot10^{13}$ ions/cm^2) bombardment conditions by Ar_{2000} and $(H_2O)_{7000}$ clusters. During prolonged bombardment the number of projectiles required to sputter (and detect) a single trehalose $[M+H]^+$ ion increases about three times in both cases.

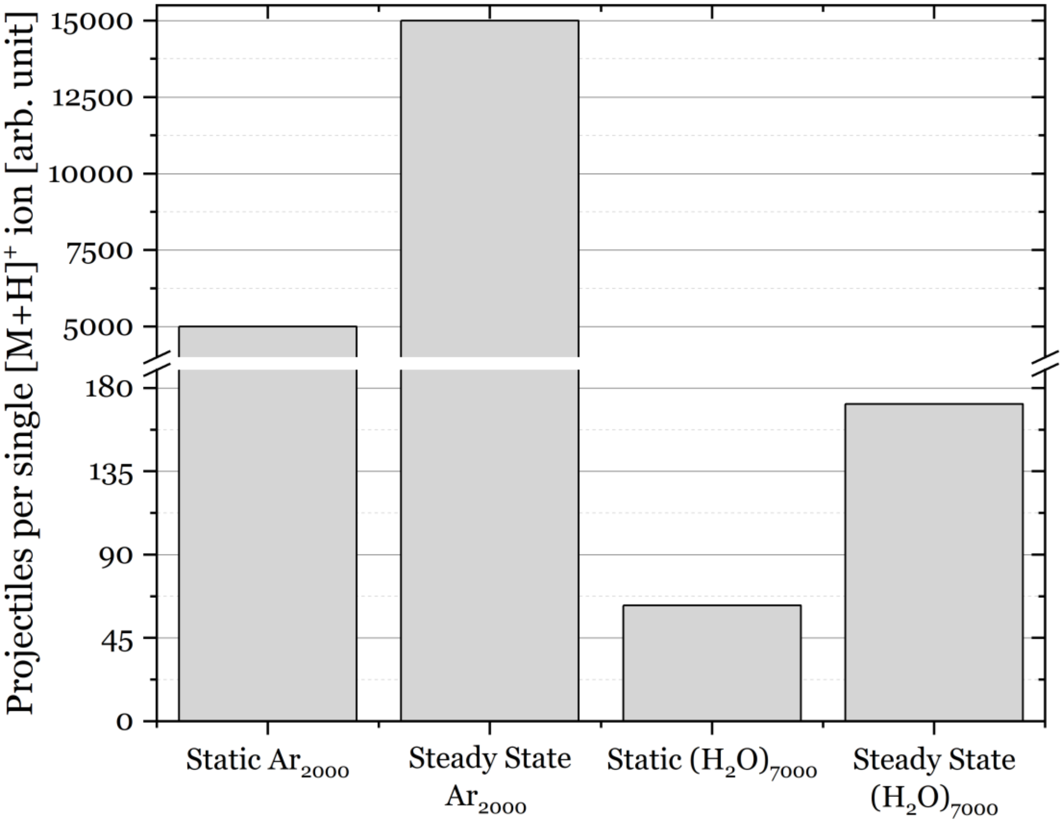

Figure S3 - Mean number of projectiles required to detect a single $[M+H]^+$ trehalose ion. Data obtained from Figure 5 in Ref. [3] by dividing the used ion dose by the number of detected ions.

Kinetic energy transfer from the projectile to the sample

Figure S4 shows how the kinetic energy is transferred from the (a, c) $(H_2O)_{4000}$ and (b, d) $(H_2O)_{15000}$ cluster projectiles to the trehalose molecules. In order to compare these two impacts, the simulation time is doubled in case of the larger cluster projectile since its velocity is about two time lower. The color scale of the visualizations is chosen in a way which highlights atoms whose kinetic energy increased.

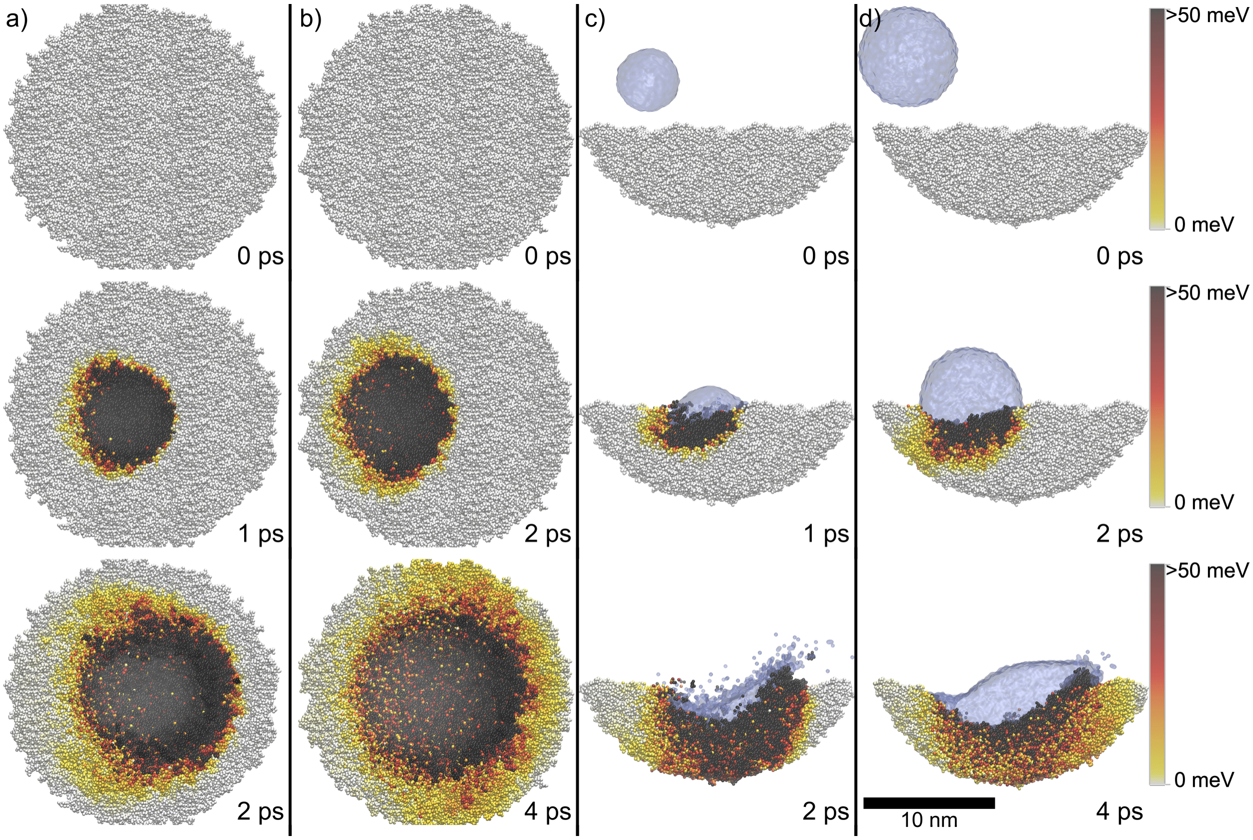

Figure S4 – Kinetic energy transfer to the trehalose sample from the (a, c) $(H_2O)_{4000}$ and (b, d) $(H_2O)_{15000}$ cluster projectiles (transparent blue) at the beginning of the impact shown in (a, b) top and (c, d) side view. The simulation times are chosen so that both projectiles travel the same distance. of the process. The kinetic energy scale is the same for all visualizations and spans from 0 to 50 meV. Some of the atoms possess kinetic energy above 50 meV. To increase visibility, only a section of the sample is shown, and the projectile molecules are hidden in (a, b). In case of (c) and (d) only a 6-nm-thick cross-section is depicted.

Influence of the minimum lifetime of the sputtered (single trehalose)-water complexes and the number of H_2O in the remaining complexes on the dependence of the number of such complexes on the kinetic energy per projectile molecule

Figure 2 in the main text shows a comparison between the amount of sputtered (single trehalose)-water complexes (blue dashed line) and the experimental signal for trehalose $[M+H]^+$ ions (black continuous line) versus kinetic energy per projectile molecule. The shape of the graph for the simulations depends on the minimum lifetime of the complexes. Additionally, some complexes are still intact in the end of the simulations, but only ones which contains at most a single H_2O molecule were included in the graph. The figures below show how these two factors impact the shape of the graph.

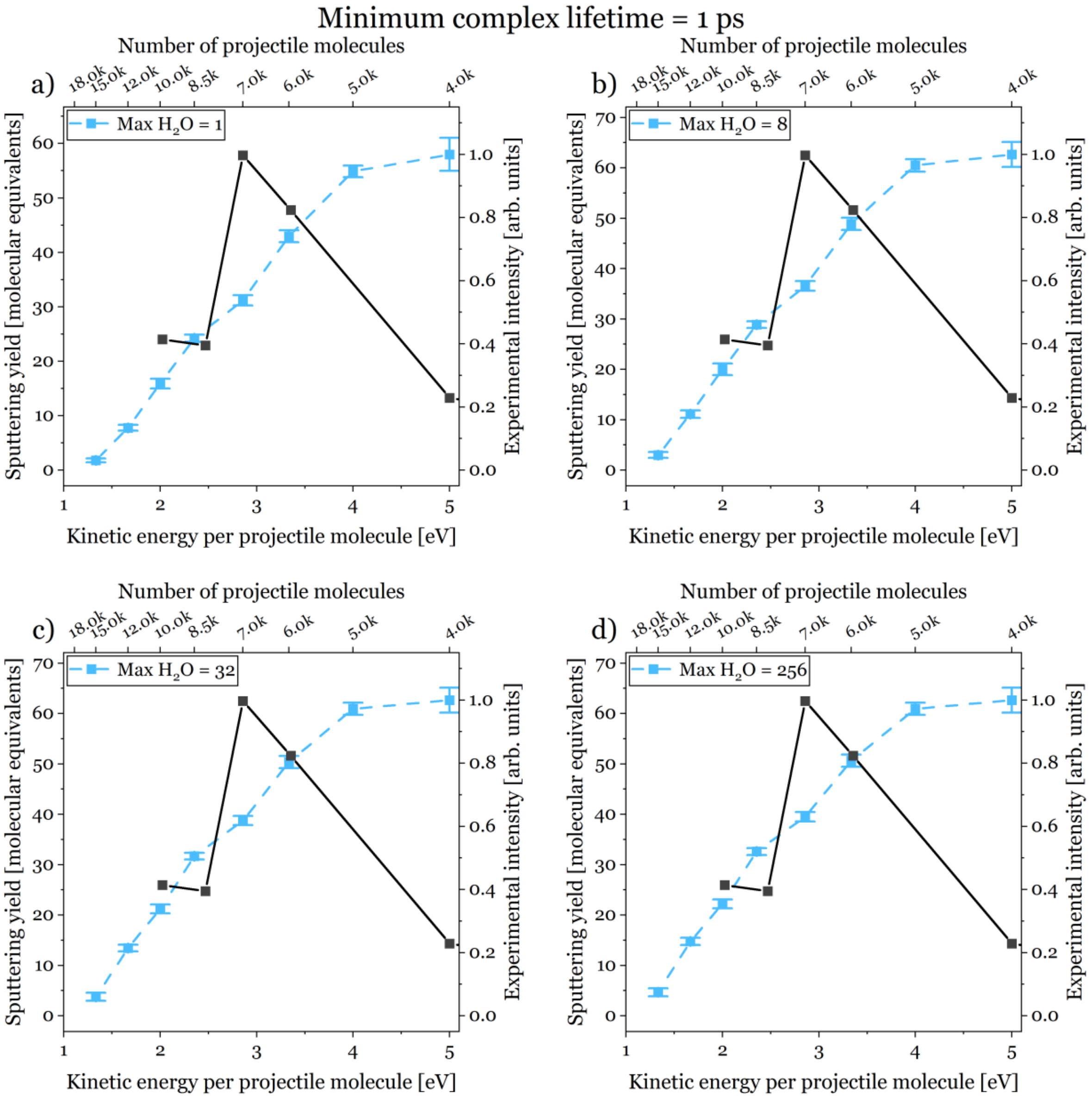

Figure S5 - Comparison between the amount of sputtered (single trehalose)-water complexes (blue dashed line) and the experimental signal for trehalose [M+H]+ ions (black continuous line) versus the kinetic energy per projectile molecule. The graphs show all complexes that were stable for at least 1 ps. Complexes with more than (a) 1, (b) 8, (c) 32, and (d) 256 H_2O molecules are excluded. The simulation data are the mean of 9 separate simulations, with the exception of results for projectiles with 8500, 7000, and 6000 water molecules where 25 simulations were performed.

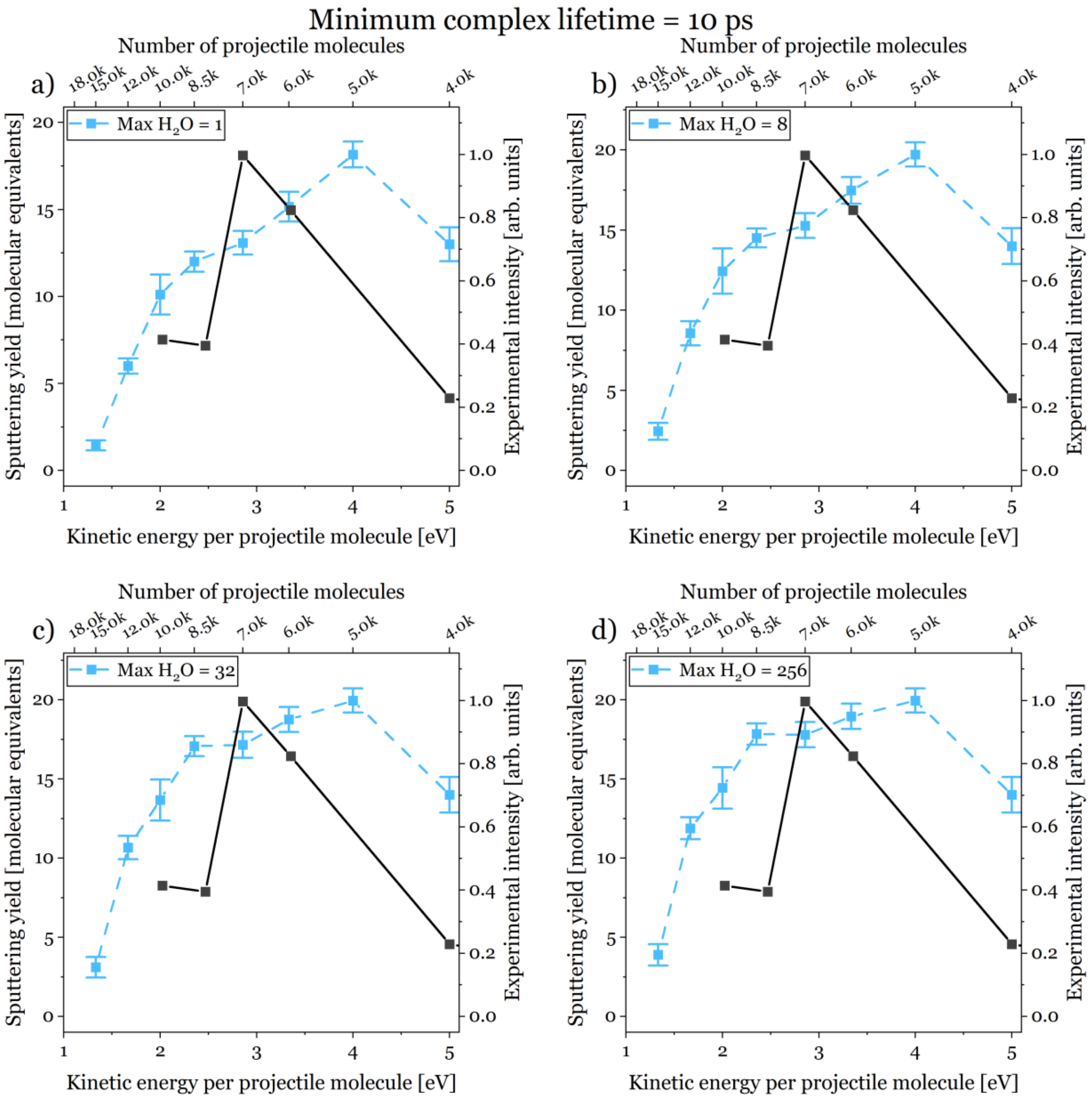

Figure S6 - Comparison between the amount of sputtered (single trehalose)-water complexes (blue dashed line) and the experimental signal for trehalose [M+H]+ ions (black continuous line) versus the kinetic energy per projectile molecule. The graphs show all complexes that were stable for at least 10 ps. Complexes with more than (a) 1, (b) 8, (c) 32, and (d) 256 H_2O molecules are excluded. The simulation data are the mean of 9 separate simulations, with the exception of results for projectiles with 8500, 7000, and 6000 water molecules where 25 simulations were performed.

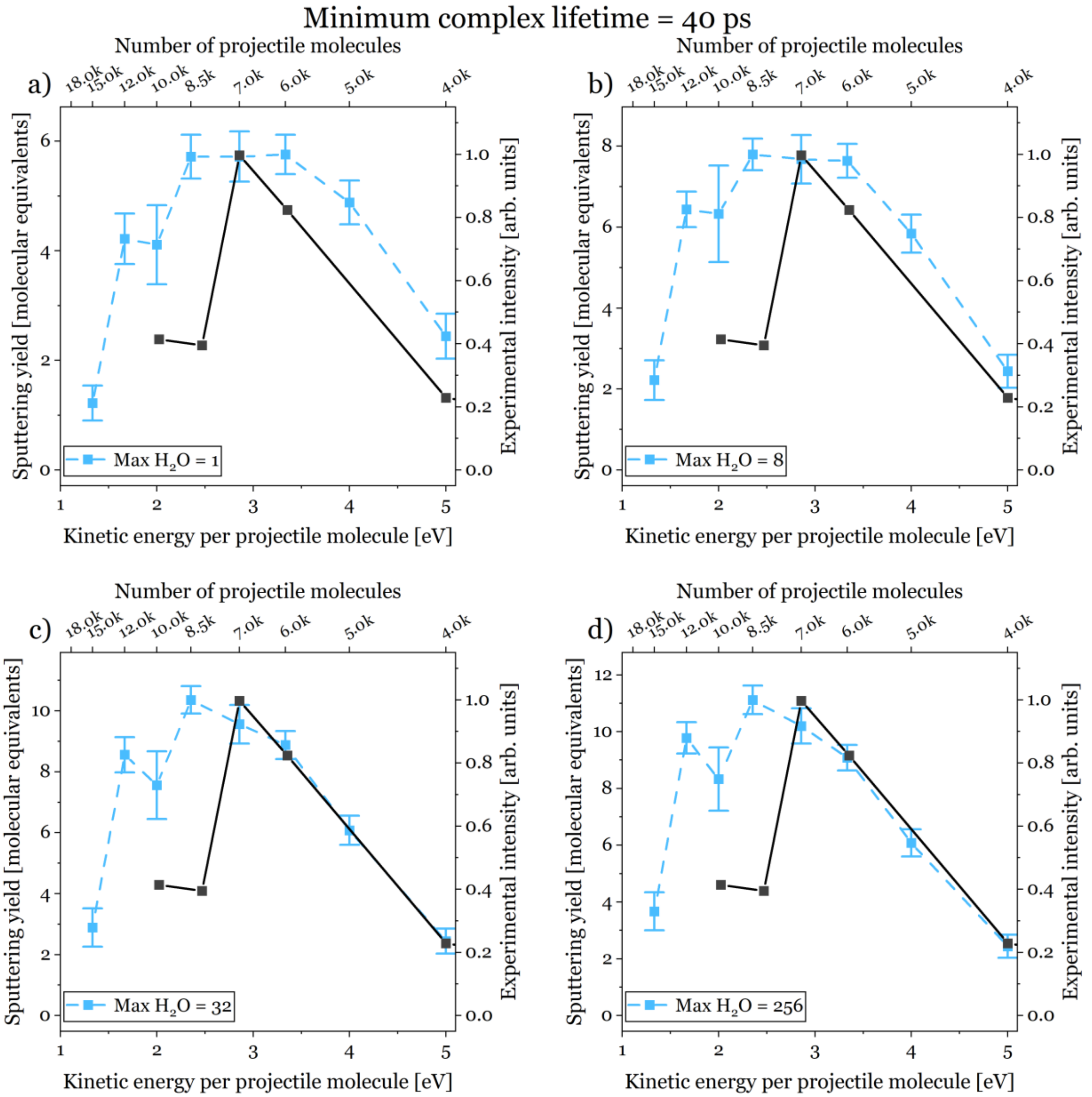

Figure S7 - Comparison between the amount of sputtered (single trehalose)-water complexes (blue dashed line) and the experimental signal for trehalose [M+H]+ ions (black continuous line) versus the kinetic energy per projectile molecule. The graphs show all complexes that were stable for at least 40 ps. Complexes with more than (a) 1, (b) 8, (c) 32, and (d) 256 H_2O molecules are excluded. The simulation data are the mean of 9 separate simulations, with the exception of results for projectiles with 8500, 7000, and 6000 water molecules where 25 simulations were performed.

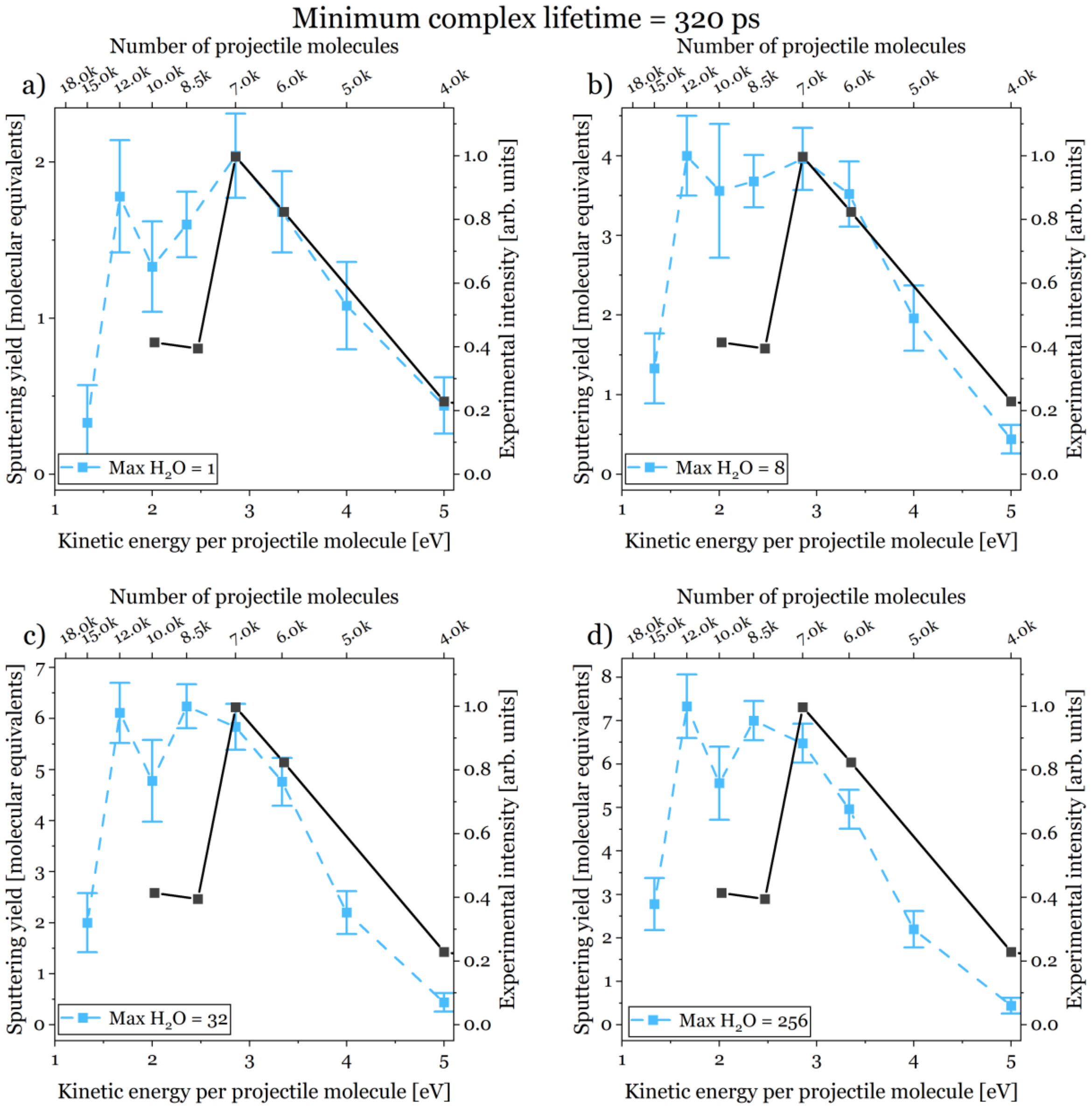

Figure S8 - Comparison between the amount of sputtered (single trehalose)-water complexes (blue dashed line) and the experimental signal for trehalose [M+H]+ ions (black continuous line) versus the kinetic energy per projectile molecule. The graphs show all complexes that were stable for at least 320 ps. Complexes with more than (a) 1, (b) 8, (c) 32, and (d) 256 H_2O molecules are excluded. The simulation data are the mean of 9 separate simulations, with the exception of results for projectiles with 8500, 7000, and 6000 water molecules where 25 simulations were performed.
